# Percutaneous tracheostomy procedures and patient results in a tertiary intensive care unit: A single-center experience

**DOI:** 10.1097/MD.0000000000041472

**Published:** 2025-02-07

**Authors:** Ayşe Vahapoğlu, Ayfer Kaya Gök, Zuhal Çavuş

**Affiliations:** a Department of Anesthesiology and Intensive Care, University of Health Sciences, Gaziosmanpaşa Training Research Hospital, Istanbul, Turkey.

**Keywords:** mortality, percutaneous dilation tracheostomy, tracheostomy timing

## Abstract

The ideal timing for tracheostomy in patients undergoing prolonged mechanical ventilation (MV) in the intensive care unit (ICU) remains controversial. The present study aimed to provide an overview of the timing of percutaneous dilation tracheostomy performed in the ICU over a 5-year period, and the effect of this procedure on 28-day mortality. The study included patients who underwent early (≤14 days) (n = 112) and late (>14 days) (n = 171) tracheostomy during their follow-up in the ICU between 2018 and 2023. It is a single-center retrospective study. The diagnoses, comorbidities, MV duration, tracheostomy timing, tracheostomy indications, tracheostomy complications, ICU length of stay, hospital length of stay, extubation attempts, mortality, time to decannulation, and ICU discharge location were determined in both tracheostomy groups and compared. The effect of tracheostomy timing on mortality risk was evaluated using multivariate Cox regression analyses. In the early tracheostomy group, MV duration, ICU hospitalization, hospital stay, and extubation attempt were lower. The 28-day intensive care mortality rates were not statistically different between the early and late tracheostomy groups. Multivariate regression analysis showed that mortality risk increased with prolonged MV and tracheostomy complications. In terms of mortality rates in palliative care, mortality in the late tracheostomy group was significantly lower than in the early tracheostomy group. The study demonstrated that the timing of tracheostomy in the ICU had no effect on mortality risk in multivariate analysis. We believe that time is not the only limiting factor when considering tracheostomy and prospective randomized studies are needed.

## 1. Introduction

Tracheostomy is a surgical procedure used in intensive care unit (ICU) patients who require prolonged mechanical ventilation (MV).^[1]^ Tracheostomy is performed by surgeons in the operating room. However, with the development of percutaneous dilatation techniques, percutaneous dilatation is now a routine procedure performed percutaneously by intensive care specialists in most ICUs.^[2]^ Percutaneous dilation tracheostomy (PDT) was first performed in 1985 by Ciaglia et al.^[3]^ PDT is an easy, fast, and minimally invasive surgical technique performed at the bedside in the ICU.

The timing of tracheostomy is classified as “early” and “late.”^[4]^ The definition of early and late tracheostomy timing varies among studies. Some studies have reported that tracheostomy is preferred between 7 and 15 days after intubation.^[5]^ Some studies suggest that early tracheostomy is associated with better outcomes than late tracheostomy^[6]^; however, there are other studies that suggest the opposite.^[7,8]^ A study comparing early and late tracheostomy found a lower mortality rate for early tracheostomy than for late tracheostomy in 7 out of 8 randomized clinical trials.^[1]^ On the other hand, a prospective observational study found no difference in 3-month mortality between early and late tracheostomy.^[9]^ The optimal timing of tracheostomy remains uncertain because of the uncertainty in the balance between the benefits and risks of tracheostomy.^[10,11]^

The advantages of early tracheostomy include minimizing oropharyngeal and laryngeal trauma, minimizing dead air space in the respiratory system, reducing breathing effort by decreasing airway resistance, improving pulmonary secretion clearance, and reducing the amount of sedation.^[12]^ It has a positive impact on the patient’s prognosis and reduces the duration of MV, and therefore the length of ICU admission and hospitalization.

The most common indications for tracheostomy are prolonged MV, upper airway obstruction, and facilitation of clearance of pulmonary secretions. As tracheostomy is an invasive procedure, there is a risk of complications such as bleeding, tracheoesophageal fistula, pneumothorax, incision infection, tracheal stenosis, and subcutaneous emphysema in the acute and long-term phases.^[13,14]^ The decision to perform tracheostomy should be evaluated on an individual patient basis.

Most patients who undergo tracheostomy in the ICU are patients who are being followed up for acute respiratory failure, coma, neuromuscular disease, or trauma.^[11]^ Patients decided to perform early or late tracheostomy after spontaneous breathing trials and weaning trials from MV according to different disease groups and severity groups.^[4]^ The development of predictive methods that can be adapted to each clinical situation will be a great advance in the decision to perform early and late tracheostomy.

The majority of ICUs were full of patients with respiratory failure requiring endotracheal intubation and MV all over the world during the coronavirus disease 2019 (COVID-19) pandemic.^[15,16]^ Tracheostomy was considered for patients requiring prolonged MV. Tracheostomy has both advantages and disadvantages, especially in COVID-19 pneumonia.^[17]^ Tracheostomy was considered a highly aerosol-generating procedure at the beginning of the pandemic^[18]^ and was also considered important for early weaning from MV and in the management of inadequate ICU beds when adequate precautions are taken. In line with previous studies,^[19,20]^ tracheostomy in patients with COVID-19 requiring prolonged MV resulted in shorter ICU and hospital stays.

Our study aimed to determine and compare the demographic, clinical characteristics, and mortality rates of patients in tertiary care ICU who underwent both early and late PDT. The authors also aimed to analyze the effect of PDT timing on the risk of 28-day mortality.

## 2. Material and methods

The present study was conducted with the approval of the ethics committee of our hospital (No: 13.09.2023/114) and included 283 patients who were followed up in the ICU and underwent bedside, early (≤14 days), and late (>14 days) tracheostomy between January 2018 and July 2023. It is a single-center retrospective study. Consent from the participants was waived due to the retrospective nature of the study. The timing of tracheostomy was decided by the intensive care specialist with an individualized approach tailored to each patient, determining patient demographics, underlying comorbidities, and organ failure. PDTs were performed in the ICU by an intensive care specialist at the bedside. Patients aged 18 years and older who underwent elective PDT after admission to the ICU were included in the study. Patient files were reviewed; age, gender, diagnosis, concomitant diseases, MV duration, tracheostomy timing, tracheostomy indications, tracheostomy complications, length of stay in ICU, length of hospital stay, place of discharge from ICU, extubation attempts, 28-day mortality, and time to decannulation were recorded. The early and late tracheostomy groups were evaluated demographically and clinically, and their effects on mortality risk were determined. Patients under 18 years of age with underlying bleeding risk, severe hypoxemia, abnormal coagulopathy, multiple organ failure, and tracheostomy before ICU admission or those tracheostomized for operative purposes were excluded from the study.

### 2.1. Statistical analysis

Quantitative data were summarized as mean ± standard deviation and median, interquartile range, and minimum–maximum values. Qualitative data were summarized as frequency and percentage, n (%), values. Tracheostomy is classified as early (14 or fewer days after intubation) and late (>14 days after intubation). The demographic and clinical data were compared between the early and late tracheostomy groups. The normality assumption for quantitative data was evaluated using the independent *t* test. Since normality was not ensured, the Mann–Whitney *U* test was used for comparisons between groups. Median survival time in ICU and palliative care was calculated by Kaplan–Meier survival analysis. The association between the demographic and clinical characteristics and mortality was analyzed using univariate and multivariate Cox regression analyses. Spline functions were used in Cox modeling to review the nonlinear relationship between mortality and tracheostomy, and the change in the hazard ratio (HR) was examined over the tracheostomy time in the multivariate model. Statistical significance was set as *P* < .05. Analyses were performed using the statistical software R version 4.1.2. The “coin” library was used for the Mann–Whitney *U* test, and the “survival,” “rms,” and “Greg” libraries were used for Cox regression (with enter method), spline functions, and graph drawing.

## 3. Results

In the study, 10 out of 293 patients who underwent tracheostomy in the ICU were transferred to an external ICU before the 28-day ICU mortality rate was reached. A total of 283 patients were included in the study, 112 of whom underwent early tracheostomy and 171 a late tracheostomy.

The mean age of patients in the early and late tracheostomy groups was 66.73 ± 19.1 and 64.43 ± 17.1 years, respectively. The male/female ratio in the early and late tracheostomy groups was 60/52 and 84/87, respectively. There were no significant differences between the 2 groups in terms of age and sex (Table 1).

**Table 1:** Comparison of demographic and clinical characteristics of patients with early and late tracheostomy.

	Tracheostomy time		*P*
	Early (≤14 d) (N = 112)	Late (>14 d) (N = 171)	
Age (yr), mean ± SD	66.73 ± 19.1	64.43 ± 17.1	.291*
Gender, n (%)			
Male	60 (53.6)	84 (49.1)	.464†
Female	52 (46.0)	87 (50.9)	
Tracheostomy timing (d), median (min–max) IQR	9 (1–14) 5	23(15–95) 10	<.001‡
Tracheostomy complication, n(%)			
None	109 (97.3)	163 (95.3)	.535§
Yes	3 (2.7)	8 (4.7)	
Decannulation, n (%)			
No	103 (92.0)	161 (94.2)	.472†
Yes	9 (8.0)	10 (5.8)	
Decannulation time (d), median (min–max) IQR	(15–396) 173 (n = 9)	105.5 (25–178) 49 (n = 10)	.624‡
Extubation attempt, n (%)			
None	96 (84.8)	106 (62.0)	<.001§
Once	14 (12.5)	41 (24.0)	
Twice	3 (2.7)	18 (10.5)	
Three times	0 (0)	5 (2.9)	
Five times	0 (0)	1 (0.6)	
Mechanic ventilation duration (d), median (min–max) IQR	22 (3–263) 24	42 (14–739) 33	<.001‡
Intensive care unit admission duration (d), median (min–max) IQR	23 (4–263) 25	46 (19–739) 32	<.001‡
Hospitalization duration (d), median (min–max) IQR	36 (4–272) 33	57 (19–739) 44	<.001‡

Quantitative data were summarized with mean ± standard deviation and median (minimum–maximum) IQR values, and qualitative data were summarized with frequency and percentage, n (%), values.

*P* < .05 considered significant.

IQR = interquartile range, max = maximum, min = minimum, SD = standard deviation.

* Independent *t* test.

†χ ^2^ Test.

‡ Mann–Whitney *U* test.

§ Fisher exact test.

The tracheostomy timing was defined as the mean time (days) between endotracheal intubation and tracheostomy. The mean time of tracheostomy in the early and late tracheostomy groups was 9 out of 23 days, respectively. A statistically significant difference was detected between the groups in terms of tracheostomy timing (*P* < .001).

Early and late tracheostomy complications included hemorrhage, pneumothorax, tracheoesophageal fistula, subcutaneous emphysema, desaturation, air leakage, and cardiac arrest.

The proportion of extubation attempts was higher in the late tracheostomy group than in the early group (38.0% and 15.2%, respectively; *P* < .001).

The MV duration is the number of MV days during hospitalization. The mean MV duration in the early and late tracheostomy groups was 22 out of 42 days, respectively. The duration of MV was longer in the late tracheostomy group than in the early tracheostomy group (*P* < .001).

The mean ICU stay in the early and late tracheostomy group was 23 out of 46 days, respectively. A longer length of ICU stay was observed in the late tracheostomy group (*P* < .001).

The mean value of hospitalization duration in the early and late tracheostomy group was 36 out of 57 days, respectively. A longer hospital stay was observed in the late tracheostomy group than in the early group (*P* < .001).

The diagnoses of the patients in the early tracheostomy group were acute respiratory failure in 59 (52.7%), brain injury in 44 (39.3%), and sepsis in 17 (15.2%). The diagnoses of the patients in the late tracheostomy group were acute respiratory failure in 106 (62.05%), brain injury in 49 (28.7%), and sepsis in 30 (17.5%) (Supplementary Table S1, Supplemental Digital Content, http://links.lww.com/MD/O355).

The comorbidities of the patients in the early tracheostomy group were neurological diseases in 44 (39.3%) patients, respiratory tract infection in 16 (14.3%), heart disease in 51 (45.5%), diabetes in 19 (17.0%), malignancy in 19 (17.0%), and others in 11 (9.9%). The comorbidities of the patients in the late tracheostomy group were neurological diseases in 54 (31.6%) patients, respiratory tract infection in 36 (21.1%), heart disease in 86 (50.3%), diabetes in 46 (26.9%), malignancy in 18 (10.5%), and others in 15 (8.8%). No statistical difference was found between the early and late tracheostomy groups in terms of diagnosis and comorbidities (Supplementary Table S1, Supplemental Digital Content, http://links.lww.com/MD/O355).

**Figure 1.**
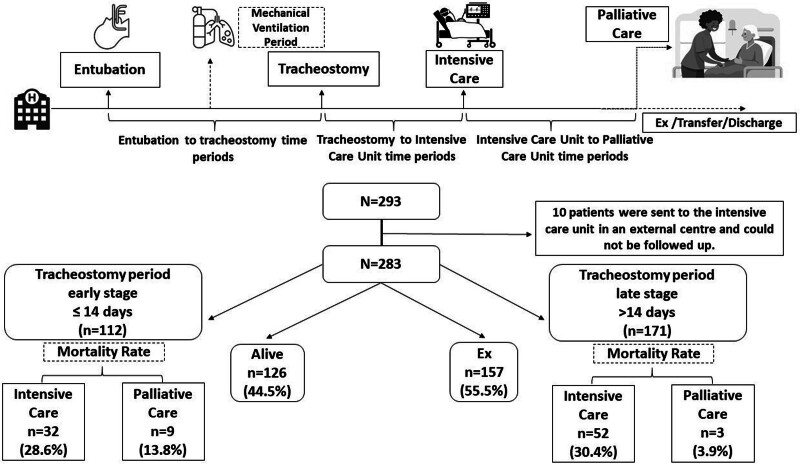
Abstract flowchart for study design.

Patients who were followed up in the ICU were followed up by a palliative care unit (PCU) integrated into the ICU when their acute ICU needs decreased, in order to continue their medical treatment, to facilitate the transfer of patients home, and to provide cost-effective service. MV follow-up of patients in PCU continued with homewent. The palliative care team continued the follow-up until the patient was discharged from the hospital. Of the 141 patients transferred to the PCU, 65 underwent early tracheostomy and 76 underwent late tracheostomy, and 28-day mortality was calculated as 12 (8.5%). There was no difference in early (≤14 days) and late (>14 days) ICU mortality rates between the early and late tracheostomy groups (28.6% vs 30.4%) (*P* = .741). In the PCU mortality was significantly lower in the late tracheostomy group than in the early tracheostomy group (13.8% vs 3.9%) (*P* = .036). When comparing the mortality rates in the ICU and the PCU, the mortality rate in the PCU was significantly lower in the group with late tracheostomy (*P* = .016) (Table 2, Fig. 1).

**Table 2:** Comparison of mortality in intensive care unit and palliative care during tracheostomy periods.

Mortality	Subgroups	Tracheostomy period		Total (%)	*P*
		Early (≤14 d)	Late (>14 d)		
		n (%)			
ICU					
	Ex	32 (28.6)	52 (30.4)	84 (29.7)	.741
	Live	80 (71.4)	119 (69.6)	199 (70.3)	
Palliative care					
	Ex	9 (13.8)	3 (3.9)	12 (8.5)	.036
	Live	56 (86.2)	73 (96.1)	129 (91.5)	
ICU versus Palliative care (Ex)					
	ICU	32 (78.1)	52 (94.6)	84 (29.7)	.016
	Palliative	9 (21.9)	3 (5.4)	12 (8.5)	

Qualitative data were summarized with frequency and percentage, n (%), values.

Pearson χ^2^ test used and *P* < .05 considered significant.

Ex = exitus, ICU = intensive care unit.

The HRs of the parameters influencing time-dependent mortality on the wards were assessed with Cox regression analysis using univariate and multivariate regression analysis (Table 3). Univariate analysis in the ICU revealed that brain injury at diagnosis (HR: 1.661, 95% confidence interval [CI]: 1.006–2.747, *P* = .047), prolonged MV (HR: 1.075, 95% CI: 1.057–1.094, *P* < .001), and tracheostomy complication (HR: 2.841, 95% CI: 1.372–5.882, *P* = .005) significantly increased the risk of mortality. Multivariate regression analysis showed that the risk of mortality increased with prolonged MV (HR: 1.078, 95% CI: 1.058–1.097, *P* < .001) and with tracheostomy complications (HR: 3.205, 95% CI: 1.527–6.711, *P* = .002) (Table 3).

**Table 3:** Univariate and multivariate Cox regression findings.

Variables	ICU				Palliative care			
	Univariate model		Multivariate model		Univariate model		Multivariate model	
	HR (95% CI)	*P*	HR* (95% CI)	*P*	HR (95% CI)	*P*	HR* (95% CI)	*P*
Age (yr)	1.009 (0.997–1.022)	.149	–	–	1.083 (1.018–1.153)	.012	1.060 (0.994–1.129)	.076
Gender								
Male versus female	1.013 (0.660–1.554)	.954	–	–	1.045 (0.319–3.425)	.942	–	–
Comorbidity								
Yes versus no	1.321 (0.755–2.310)	.329	–	–	2.809 (0.359–21.739)	.325	–	–
Diagnosis								
Respiratory failure (yes vs no)	1.425 (0.909–2.237)	.123	–	–	1.968 (0.576–6.727)	.280	–	–
Brain damage (yes vs no)	1.661 (1.006–2.747)	.047	1.546 (0.934–2.557)	.090	1.093 (0.320–3.739)	.887	–	–
Sepsis (yes vs no)	1.161 (0.674–2.004)	.590	–	–	4.000 (1.166–13.699)	.028	2.392 (0.660–8.696)	.184
Tracheostomy time								
Late (>14 d) versus early (≤14 d)	1.149 (0.739–1.786)	.537	–	–	5.747 (1.244–26.316)	.025	3.459 (0.715–16.725)	.123
Mechanic ventilation duration (d)	1.075 (1.057–1.094)	<.001	1.078 (1.058–1.097)	<.001	1.045 (0.997–1.097)	.065	–	–
Tracheostomy complication								
Yes versus no	2.841 (1.372–5.882)	.005	3.205 (1.527–6.711)	.002	20.501 (0.000–NA)	.808	–	–
Extubation trial								
≥2 Versus no	1.709 (0.688–4.249)	.249	–	–	2.326 (0.145–37.037)	.550	–	–
≥2 Versus 1	1.678 (0.615–4.582)	.312	–	–	1.012 (0.128–8.000)	.991	–	–

CI = confidence interval, HR = hazard ratio, ICU = intensive care unit.

* Adjusted hazard ratio.

In the PCU, age (HR: 1.083, 95% CI: 1.018–1.153, *P* = .012), the presence of sepsis (HR: 4.000, 95% CI: 1.166–13.699, *P* = .028), and a tracheostomy duration of >14 days (HR: 5.747, 95% CI: 1.244–26.316) increased the risk of mortality in the univariate analysis. No significant correlation was found in the multivariate regression analysis performed with the significant parameters (Table 3).

**Figure 2.**
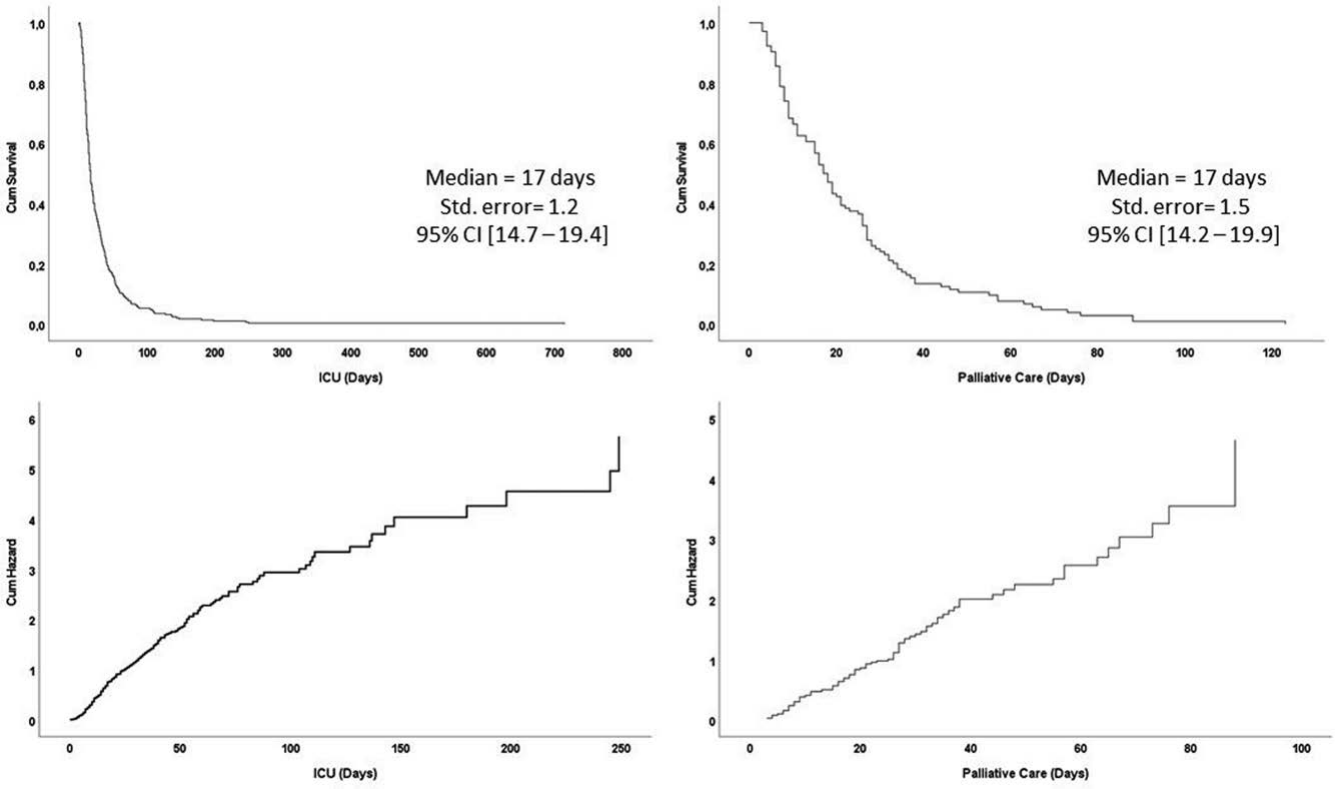
Kaplan–Meier survival and Cox regression analysis. CI = confidence interval, ICU = intensive care unit.

Survival times in ICU and PCUs were analyzed. The median survival time in ICU was 17 days (95% CI: 14.7–19.4), while the median survival time in PCU was 17 days (95% CI: 14.2–19.9). In ICU, the cumulative HR was 5 at 250 days, while in PCU, the cumulative HR was close to 4 at 90 days (Fig. 2).

In the present study, tracheostomy was performed in 31 patients with COVID-19 positive who underwent MV. The mean age of the group without COVID-19 and the group with COVID-19 was 64.65 and 70.90 years, respectively. There were no differences between the 2 groups in terms of age, sex, time of tracheostomy, tracheostomy complications, MV, length of hospital stay, and ICU stay (Table 4).

**Table 4:** Comparison of those with and without COVID-19 in terms of demographic and clinical characteristics.

Variables	COVID-19		*P*
	None	Yes	
	(n = 252)	(n = 31)	
Age (yr), mean ± SD	64.65 ± 18.5	70.90 ± 11.1	.067*
Gender, n (%)			
Male	130 (51.6)	14 (45.2)	.499†
Female	122 (48.4)	17 (54.8)	
Tracheostomy timing (d), median (min–max) IQR	17 (1–95) 14	21 (5–37) 16	.203‡
Tracheostomy time, n (%)			
Early (≤14 d)	102 (40.5)	10 (32.3)	.377†
Late (>14 d)	150 (59.5)	21 (67.7)	
Tracheostomy complication, n (%)			
None	244 (96.8)	28 (90.3)	.107§
Yes	8 (3.2)	3 (9.7)	
Decannulation, n (%)			
None	233 (92.5)	31 (100)	.243§
Yes	19 (7.5)	0 (0)	
Decannulation time (d) (n = 19) Median (min–max) IQR	0 (0–396) 0	–	
Extubation attempt, n (%)			
0	172 (68.3)	29 (93.5)∥	.040§
One time	55 (21.8)∥	0 (0)	
Two times	19 (7.5)	2 (6.5)	
Three times	5 (2.0)	0 (0)	
Five times	1 (0.4)	0 (0)	
Mechanic ventilation duration (d), median (min–max) IQR	35 (3–739) 32	33 (7–263) 23	.616‡
Intensive care unit admission duration (d), median (min–max) IQR	38 (4–739) 35	38 (7–263) 23	.735‡
Hospitalization duration (d), median (min–max) IQR	50 (4–739) 46	41(7–272) 42	.241‡
Mortality, n (%)			
Live	116 (46.0)	10 (32.3)	.145†
Exitus	136 (54.0)	21 (67.7)	

Quantitative data were summarized with mean ± standard deviation and median (minimum–maximum) IQR values, and qualitative data were summarized with frequency and percentage, n (%), values.

COVID-19 = coronavirus disease 2019, IQR = interquartile range, max = maximum, min = minimum, SD = standard deviation.

* Independent *t* test.

†χ ^2^ Test.

‡ Mann–Whitney *U* test.

§ Fisher exact test.

∥ *P* < .05 considered significant.

None of the patients with COVID-19 had decannulation. No extubation attempts were performed in 93.5% of the patients with COVID-19 and 6.5% had 2 extubation attempts. Since the *P* value was .040, there was a borderline association between the number of extubation attempts and COVID-19 (Table 4).

## 4. Discussion

The experience of elective PDT performed at the bedside in the ICU and the effect of tracheostomy timing on 28-day mortality was assessed in this study. No significant association was found between tracheostomy timing and 28-day mortality risk in multivariate analysis.

The number of ICU patients requiring prolonged MV and tracheostomy has increased in recent years. The timing of tracheostomy is a constant variable that depends on the experience of the physician and the standard procedures of the ICU.^[4]^

The benefits of tracheostomy are that it can significantly shorten the duration of MV, ICU, and hospital stay.^[6,21,22]^ Therefore, tracheostomies are expected to improve patient survival.^[23]^ No significant association was found between the timing of tracheostomy and mortality risk in our study. Some authors have reported no difference between early and late tracheostomy in terms of mortality.^[24-26]^ Siempos et al^[27]^ and Griffiths et al.^[28]^ compared early tracheostomy with late tracheostomy, and found that it did not improve survival.

Chen et al focused on the factors influencing the outcome of tracheostomy timing in a study with a large sample size of 1209 patients and found that early tracheostomy could significantly shorten the duration of MV, ICU, and hospital stay.^[4]^ In this study, early tracheostomy was associated with shorter MV duration.

Khammas et al^[29]^ found that there was no statistically significant difference in the total ICU length of stay in the early tracheostomy group when compared with the late tracheostomy group. Bösel et al,^[30]^ Young et al,^[25]^ and Barquist et al^[8]^ found that the timing of tracheostomy did not affect the length of ICU stay. However, early tracheostomy was associated with shorter ICU and hospital stays in this study. Liu et al^[31]^ found a reduction in ICU length of stay in the early tracheostomy group in a recent meta-analysis of 11 studies; however, they did not find any difference in in-hospital mortality. In our study, the length of stay in the ICU and the duration of MV were shorter in the early tracheostomy group than in the late tracheostomy group. These results seem to be consistent with the literature. McCredie et al^[32]^ found that early tracheostomy was associated with a reduction in MV and ICU length of stay when compared with late tracheostomy but not with a reduction in short-term mortality.

The most common indications detected in previous studies were respiratory diseases, head-neck tumors, and jaw-brain trauma.^[33]^ The most common indications for tracheostomy were multiple, including pulmonary secretion clearance, prolonged intubation, failed extubation, upper airway obstruction, airway protection, and anticipated difficult intubation in this study.

Patients on MV who were admitted to the ICU represent a heterogeneous disease group. The most common concomitant diseases in patients with tracheostomy were respiratory infections and neurological diseases. In this study, it was observed that patients with brain injury required long-term MV due to decreased consciousness, inability to protect the airways, and inability to clear secretions, and benefited from early tracheostomy.

PDT is a safe procedure that can be performed at the bedside with a low complication rate.^[34]^ Fernandez-Bussy et al^[35]^ listed early and late complications after tracheostomy as pneumothorax, bleeding, subcutaneous emphysema, wound infection, tracheal stenosis, esophageal injury, and tracheoesophageal fistula. The most common complication of PDT is bleeding, which is usually self-limited or controlled with simple interventions.^[36]^ Bleeding was also the most common complication in our study. In addition, multivariate regression analysis showed that prolonged MV and tracheostomy complications increased the risk of mortality.

Global guidelines have not reached a consensus on the optimal timing and outcomes of tracheostomy in patients with COVID-19. Since viral infectivity ended 21 days after the onset of symptoms in the majority of patients, tracheostomy was performed after 21 days. In our study, tracheostomy was performed in COVID-19 patients 21 days after intubation in accordance with the guidelines.^[37]^ In cases where tracheostomy should be performed earlier for reasons such as the need for pulmonary secretion clearance by the intensivist or to reduce sedation, the timing of tracheostomy was performed earlier.

This study showed that there was no association between age, sex, timing of tracheostomy, tracheostomy complications, duration of MV, ICU stay, length of hospital stay, and mortality in patients with COVID-19 who underwent tracheostomy.

In the present study, there was no significant difference in mortality between COVID-19 and non-COVID-19 tracheostomized groups indicating that tracheostomy is safe to perform in patients with COVID-19. We believe that for patients with COVID-19 with respiratory failure requiring prolonged MV, tracheostomy is an integral part of the patient management plan.

Disease severity and state of consciousness are factors that affect early extubation.^[38]^ Severe disease reduces the chance of early extubation; therefore, intensive care physicians may favor early tracheostomy. Since extubation attempts could not be made in patients with COVID-19 who were difficult to wean from MV in the ICU in this study, a statistically significant association was detected between the groups.

Rules for the discharge of patients from intensive care are necessary for the efficient use of ICUs.^[39,40]^ Establishing discharge criteria and improving the quality of life of patients undergoing tracheostomy may vary according to the social and economic status of the patient and caregiver and whether end-of-life decisions are implemented in countries.^[11]^ In our country, there is no legal regulation for end-of-life decisions.

As patients with tracheostomies place an additional burden on the ICUs, we work together with the PCU to plan their discharge from the ICU. The proportion of patients referred to the PCU appears to be high in the study. Our patients were followed by a PCU integrated into the ICU. MV monitoring of patients in PCU, with homewent continued. The patient was followed up by the palliative care team until discharge from the hospital. The structure and implementation of the palliative care team may vary from 1 institution to another. PCUs integrated into the ICU are necessary services where patients with chronic diseases or complex medical conditions are transferred when their acute intensive care needs are reduced, where long-term medical treatment continues, to facilitate the transfer of patients home and to provide cost-effective services.

In the last 2 decades, PCU has emerged as an essential component of ICUs. Rubio et al^[41]^ performed a tracheostomy in 133 patients in their 7-year tracheostomy experience and used an intermediate ICU for patient discharge. We think that the higher tracheostomy rate in the 5-year period in our study may be due to the different end-of-life decisions. Ma et al^[42]^ observed a reduction in hospital and ICU length of stay with palliative care in their study. In our study, patients with early tracheostomy had high mortality in PCU. Early tracheostomy may be associated with short ICU stays and high mortality in PCU.

- The optimal timing of PDT is controversial and no significant association was found between the timing of tracheostomy and the risk of mortality.
- Patients who underwent early tracheostomy were associated with shorter MV duration, ICU, and hospital length of stay.
- The most common indications for PDT were multiple, including pulmonary secretion clearance, prolonged intubation, failed extubation, upper airway obstruction, airway protection, and anticipated difficult intubation.
- The most common comorbidities in patients with PDT were respiratory tract infections and neurological diseases.
- The most common complication of PDT is bleeding, which is usually self-limiting or controlled with simple interventions.
- Our data suggest that PCUs may be the solution for early discharge of patients with tracheostomy from the ICU to home.

## 5. Limitations

Our study has some limitations. It was a single-center, retrospective study, and the decision regarding the timing of tracheostomy was based on the opinion of the attending physician and the clinical condition of the patient. Therefore, possible interpretation bias may have affected the results of the study parameters. In this study, no association was found between tracheostomy timing and mortality risk in multivariate analysis. However, further multicenter, randomized controlled studies are needed in this regard.

## 6. Conclusion

Tracheostomy is indicated for ICU patients with severe disease and prolonged orotracheal intubation. Patients undergoing tracheostomy comprise a heterogeneous group of critically ill patients. Consequently, we found an association between early tracheostomy and ICU length of stay, MV duration, and length of hospital stay. However, no significant association was found between the timing of tracheostomy and mortality risk in the multivariate analysis. The increased mortality rate for early tracheostomy in palliative care patients may be related to the inadequate development of standards of care for patients with tracheostomy. Rules for the discharge of patients with tracheostomy from the ICU should be established. Future studies should focus on improving the long-term quality of life of patients with tracheostomy. Randomized controlled trials exploring the tracheostomy timing on patients’ outcomes are warranted and would provide evidence-based recommendations on this issue.

## Acknowledgments

The authors would like to thank all the respondents in this study.

## Author contributions

**Conceptualization:** Ayşe Vahapoğlu, Ayfer Kaya Gök.

**Investigation:** Ayşe Vahapoğlu, Zuhal Çavuş.

**Validation:** Ayşe Vahapoğlu.

**Writing – original draft:** Ayşe Vahapoğlu.

**Writing – review & editing:** Ayşe Vahapoğlu, Ayfer Kaya Gök, Zuhal Çavuş.

**Formal analysis:** Ayfer Kaya Gök, Zuhal Çavuş.

**Methodology:** Ayfer Kaya Gök.

**Data curation:** Zuhal Çavuş.

## Supplementary Material


