# Supplementary material for: Percutaneous tracheostomy procedures and patient results in a tertiary intensive care unit: A single-center experience
Source: Medicine (Baltimore). 2025 Feb 7;104(6):e41472. doi: 10.1097/MD.0000000000041472 (PMC11813018; doi:10.1097/MD.0000000000041472)
Supplement: Supplementary file 1 [file medi-104-e41472-s001.docx]

**Supplemental Digital Content**

Percutaneous tracheostomy procedures and patient results in a tertiary intensive care unit: A single-center experience.

Ayşe Vahapoğlu, Ayfer Kaya Gök, Zuhal Çavuş

**e- Supplementary Table 1.** Comparison of comorbidity and diagnosis parameters among tracheostomy periods.

| **Variables** | **Subgroups** | **Tracheostomy Period** | | **Total (%)** | **p** |
| --- | --- | --- | --- | --- | --- |
|  |  | **Early (≤14 days)** | **Late (>14 days)** |  |  |
|  |  | **n(%)** | |  |  |
| Neurological diseases |  |  |  |  |  |
|  | No | 68(60.7) | 117(68.4) | 185(65.4) | 0.183^§^ |
|  | Yes | 44(39.3) | 54(31.6) | 98(34.6) |  |
| Malignancy |  |  |  |  |  |
|  | No | 93(83.0) | 153(89.5) | 246(86.9) | 0.116^§^ |
|  | Yes | 19(17.0) | 18(10.5) | 37(13.1) |  |
| Respiratory tract infection |  |  |  |  |  |
|  | No | 96(85.7) | 135(78.9) | 231(81.6) | 0.151^§^ |
|  | Yes | 16(14.3) | 36(21.1) | 52(18.4) |  |
| Heart disease |  |  |  |  |  |
|  | No | 61(54.5) | 85(49.7) | 146(51.6) | 0.434^§^ |
|  | Yes | 51(45.5) | 86(50.3) | 137(48.4) |  |
| Diabetes |  |  |  |  |  |
|  | No | 93(83.0) | 125(73.1) | 218(77.0) | 0.052^§^ |
|  | Yes | 19(17.0) | 46(26.9) | 65(23.0) |  |
| Psychiatric Illness |  |  |  |  |  |
|  | No | 108(96.4) | 167(97.7) | 275(97.2) | 0.717^£^ |
|  | Yes | 4(3.6) | 4(2.3) | 8(2.8) |  |
| Kidney Disease |  |  |  |  |  |
|  | No | 106(94.6) | 165(96.5) | 271(95.8) | 0.550^£^ |
|  | Yes | 6(5.4) | 6(3.5) | 12(4.2) |  |
| Chronic Alcoholism |  |  |  |  |  |
|  | No | 111(99.1) | 168(98.2) | 279(98.6) | 1.000^£^ |
|  | Yes | 1(0.9) | 3(1.8) | 4(1.4) |  |
| Substance Use |  |  |  |  |  |
|  | No | 112(100) | 169(98.8) | 281(99.3) | 0.520^£^ |
|  | Yes | 0(0) | 2(1.2) | 2(0.7) |  |
| Respiratory failure |  |  |  |  |  |
|  | No | 53(47.3) | 65(38.0) | 118(41.7) | 0.120^§^ |
|  | Yes | 59(52.7) | 106(62.0) | 165(58.3) |  |
| Brain injury |  |  |  |  |  |
|  | No | 68(60.7) | 122(71.3) | 190(67.1) | 0.063^§^ |
|  | Yes | 44(39.3) | 49(28.7) | 93(32.9) |  |
| Sepsis |  |  |  |  |  |
|  | No | 95(84.8) | 141(82.5) | 236(83.4) | 0.601^§^ |
|  | Yes | 17(15.2) | 30(17.5) | 47(16.6) |  |

Qualitative data were summarized with frequency and percentage, n(%), values.

§: Pearson’s Chi-square test, £: Fisher’s Exact test.
